# A Brief Review of Graphene-Based Biosensors Developed for Rapid Detection of COVID-19 Biomarkers

**DOI:** 10.3390/bios13030307

**Published:** 2023-02-22

**Authors:** Narendhar Chandrasekar, Ramachandran Balaji, Ramaswamy Sandeep Perala, Nik Zulkarnine Nik Humaidi, Kirubanandan Shanmugam, Ying-Chih Liao, Michael Taeyoung Hwang, Saravanan Govindaraju

**Affiliations:** 1 Department of BioNano Technology, Gachon University, 1342 Seongnam-Daero, Sujeong-Gu, Seongnam-si 13120, Republic of Korea; 2 Department of Chemical Engineering, National Taiwan University, Taipei 10617, Taiwan; 3 Saveetha School of Engineering, Saveetha Institute of Medical and Technical Sciences (SIMATS), Thandalam, Chennai 602105, India; 4 Department of Bio Engineering, Saveetha School of Engineering, Saveetha Institute of Medical and Technical Sciences (SIMATS), Thandalam, Chennai 602105, India

**Keywords:** COVID-19, antigens, SARS, complementary DNA, protein–DNA

## Abstract

The prevalence of mutated species of COVID-19 antigens has provided a strong impetus for identifying a cost-effective, rapid and facile strategy for identifying the viral loads in public places. The ever-changing genetic make-up of SARS-CoV-2 posts a significant challenfge for the research community to identify a robust mechanism to target, bind and confirm the presence of a viral load before it spreads. Synthetic DNA constructs are a novel strategy to design complementary DNA sequences specific for antigens of interest as in this review’s case SARS-CoV-2 antigens. Small molecules, complementary DNA and protein–DNA complexes have been known to target analytes in minimal concentrations. This phenomenon can be exploited by nanomaterials which have unique electronic properties such as ballistic conduction. Graphene is one such candidate for designing a device with a very low LOD in the order of zeptomolar and attomolar concentrations. Surface modification will be the significant aspect of the device which needs to have a high degree of sensitivity at the same time as providing a rapid signaling mechanism.

## 1. Introduction

In the pandemic era, the uncontrollable spread of COVID-19 has necessitated the development of a novel sensor which can efficiently detect the virus in its current form as well as in future mutated forms. Early detection can be performed by the rapid analytical techniques using graphene-based materials. The COVID-19 antigens are among the best targeting sites for early detection of viral infection in the human system. This can be performed via the reliable and rapid process of a label-free diagnostic method coupled with synthetic DNA constructs. Generally, the synthesis of DNA constructs and fabrication into synthons, genes, biological circuits and construction of genomes maps via chemical synthesis methods are emerging research area in the field of molecular diagnostic technology. Alternatively, these DNA constructs can be used in the low-cost rapid diagnostic methods for various viral infections [1]. Since December 2019, COVID-19 was declared as a pandemic by the WHO, urging the development of highly sensitive biomarker-based detection methods capable of operating in robust conditions for detection of COVID-19 and their variants in biofluids [2].

The rapid sensing technique for detecting COVID-19 biomarkers in all phases of infection stages is a challenging task in molecular virology and provides a platform for controlling the infection in human. The diagnostic method for COVID-19 can be broadly divided into: (1) detection and investigation of the nucleic acid genome of the SARS-CoV-2 via qualitative reverse transcriptase polymerase chain reaction (qRT-PCR) and loop-mediated isothermal amplification (LAMP), (2) detection of viral antigens/proteins by the enzyme-linked immunosorbent assay (ELISA) or lateral flow assay (LFA), (3) studying antibodies produced by the human immune system due to viral infections. The US-FDA (United States–Food and Drug Administration) authenticated the above methods for commercialization and they are currently in practice. However, the Abbott-based COVID-19 diagnostic method gave false responses even at the early stage of coronavirus infection due to poor sensitivity and specificity problems. This occurred due to the low availability of biomarkers such as viral RNA, viral proteins and antibodies in the serum of the human blood samples collected from the coronavirus patients.

RT-PCR is now the most desired standard technique for detection of COVID-19. RT-PCR is an accurate test for diagnosis of COVID-19 in humans. However, it takes longer, up to days, to obtain results since skilled personnel are required to operate the instrument. In the design and engineering of a diagnostic method for detection of COVID-19, the bottlenecks in analytical techniques to assay COVID-19-associated biomarkers in saliva and blood/plasma/serum and/or nasal swabs are: (1) the inability to assay different types of biomolecules in one instrument—for example, the RT-PCR and LAMP techniques are capable of assaying viral nucleic acids but not proteins; (2) ELISA and LFA only detect proteins but not RNAs; and (3) general inadequacy in assay sensitivity/limit of detections to quantify a low abundance of biomarkers while retaining high specificity and accuracy [3].

**Figure 1:**
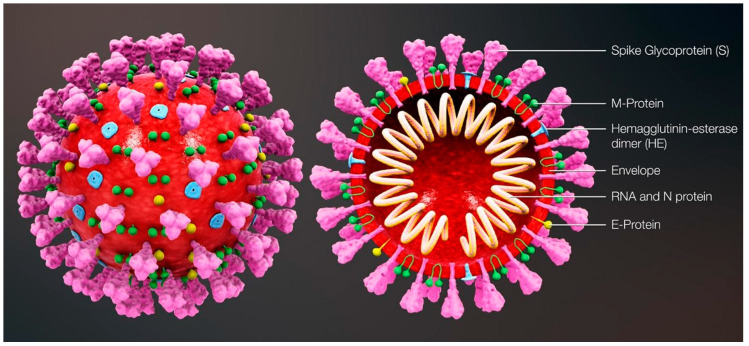
Structure and components of SARS-CoV-2. Reproduced with permission from Ref. [1]. Copyright 2022 Elsevier.

The most commonly relied upon COVID-19 sensing techniques ELISA, PCR and RT-PCR are reliable but require expansive instrumentation, training and time for diagnosis. PCR is predominantly used to detect specific organisms such as viruses. PCR is a very powerful technique as it can identify the residual fragments of a virus even after the disease disappears and so it has enabled rapid analysis to diagnose diseases in humans. RT-PCR is a laboratory-based investigation process which produces copies of specific DNA/RNA sequences for investigation. Enzymes belonging to RT are manipulated and used to vary a specific sequence of RNA to a matching piece of DNA. After producing numerous pieces of DNA in large quantities through DNA polymerase, it can identify a specific mRNA sequence which makes up a gene. RT-PCR can help to efficiently diagnose a disease and monitor an infection. So, in standard PCR analysis, a DNA template is amplified and a thermocycler is used; but in RT-PCR, RNA is utilized as a template and is reverse transcribed into complementary DNA. PCR is typically employed for viruses that have DNA for amplification [1]. Similarly, SARS-CoV-2 has only RNA and it can be detected through RT-PCR. Figure 1 shows the structure of and components present in SARS-CoV-2.

**Figure 2:**
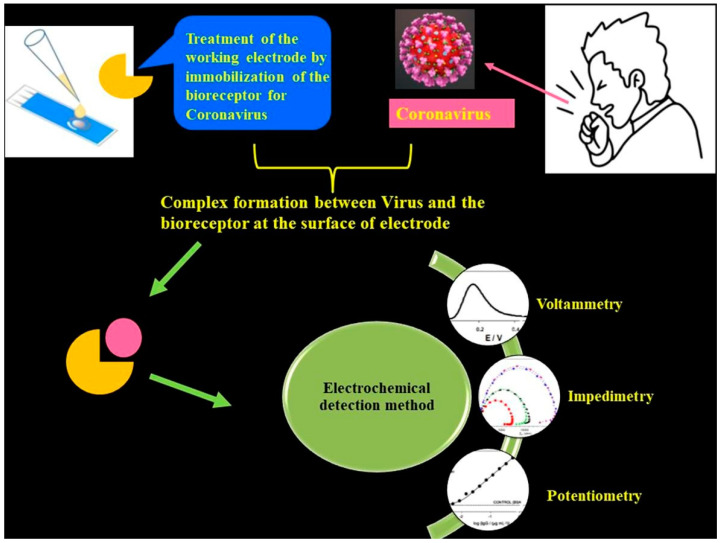
Schematic of an electrochemical COVID-19 detection technique using voltametric, impedimetric and potentiometric sensors. Reproduced with permission from Ref. [1]. Copyright 2022 Elsevier.

The aforementioned detection techniques [1,2] suffer from certain demerits, such as the need for trained and qualified personnel and also more demand for expensive chemical reagents. Importantly, traditional techniques are not suited for large-scale diagnosis in quick time frames. Although these techniques exhibit great sensitivity, sometimes they provide false positive or negative test results. It is noteworthy to mention that the sensitivity and the limit of detection (LOD) are respective to the COVID-19 viral dose and infective virus dose, which becomes an issue for sensing platforms. For RT-PCR testing of nasal samples, standard COVID-19 detection techniques provide an average LOD of 100 copies of viral RNA per mL [4]. Having said that, more than 10,000 fold the LOD can be achieved through electrochemical-based detection techniques (Figure 2).

Electrochemical detection is among the significant, low-cost and high-performance sensing techniques [2,3] which can effectively detect changes in the current, voltage or charge generated from surface interactions with a working electrode and analyte. The salient features of the electrochemical sensing platform are their user-friendly operation, low-cost fabrication, rapid detection capability and a superior LOD. These advantages can achieve the global need for diagnosing COVID-19 at larger scales. The overall performance of the electrochemical technique is improved through integration of nanomaterials into the system. Nanomaterials such as graphene family materials (GFM) can help in the successful interaction between analytes and sensors largely due to their superior specific surface area that aids in precise, rapid detection of virus biomarkers.

## 2. Significance of Synthetic DNA Constructs

The ease of access through which oligonucleotides are being synthesized by chemical methods has resulted in the construction of custom-made synthetic DNA strands or synthons being used as recognition units for biosensors. The ever-adapting SARS-CoV-2 is tough to track for sudden mutations which are specific to certain locations. However, identification of the commonly occurring nucleotide sequences in the SARS-CoV-2 antigens can be targeted by the synthetic constructs.

Batches of such synthetic DNA constructs can be fabricated by using controlled porous glass beads, wherein the oligonucleotides are grown over the glass bead surface as the feed chemicals are made to flow over the surface. Microarray-based synthesis facilitates the growth of several unique strands of DNA at the same time as enabling the methodology to produce novel and customized nucleotide pools which can be used as probes or complementary strands for target analytes such as COVID-19 antigens [1,2,3].

A single microchip can be fabricated for a variety of such oligonucleotide pools, which can then be segmented into specific sub pools so that they can be utilized to generate assembly-specific harvests. Such high-throughput fabrication of oligonucleotides can be employed to target several mutant strains or different species of microbes altogether [3,4,5].

Kosuri et al. [6] describes the scalable gene synthesis on a DNA chip from the OLS pool without distinguishing dsDNA and ssDNA, cleaved to form an oligonucleotide pool. Yellow or brown indexed primers are used to amplify separate plate sub pools with DNA to assemble different genes. Blue-colored sequences are assembly specific and are used to amplify assembly sub pools that have the DNA required to make one unique gene. The primer sequences are cleaved, forming dsDNA by using type IIS restriction enzymes; or forming ssDNA by DpnII/USER/λ exonuclease. Construction primers are employed to proceed with assembly PCR reaction to build one unique gene from each assembly sub pool. The assembled products are cloned and validated by enzyme-mediated correction.

## 3. The Surface Specificity of Graphene

Surface functionalization of graphene enhances its selectivity towards biomolecules of interest during device construction. This can be achieved through covalent and non-covalent surface modifications of graphene. In general, graphene is hydrophobic [7,8] and interacts well with organic molecules containing aromatic units through π–π interactions, especially in the basal area of graphene. This makes the adsorption of molecules on graphene’s surface non-specific without any functionalization [9]. To anchor biorecognition molecules, a linker molecule is used. The linker has aromatic components, commonly pyrene groups or any molecule that governs the π-moiety in its structure. Biorecognition units which are meant to be anchored on the surface via linker have to be specific towards the target molecule—in this review’s case, COVID-19 antigens. Functionalization of the graphene surface can be achieved by attaching organic molecules comprising aromatic moieties through Van der Waals interaction [7,8,10,11]. The specificity of the graphene surface can be engineered by introducing linker molecules, which allows specific molecules to adhere; however, there are still chances for non-specific binding to occur [9,11] due to surface coverage of linker molecules, thereby leading to free sites on graphene that could invite non-specific interactions.

## 4. Passivation and Functionalization of Surface

**Figure 3:**
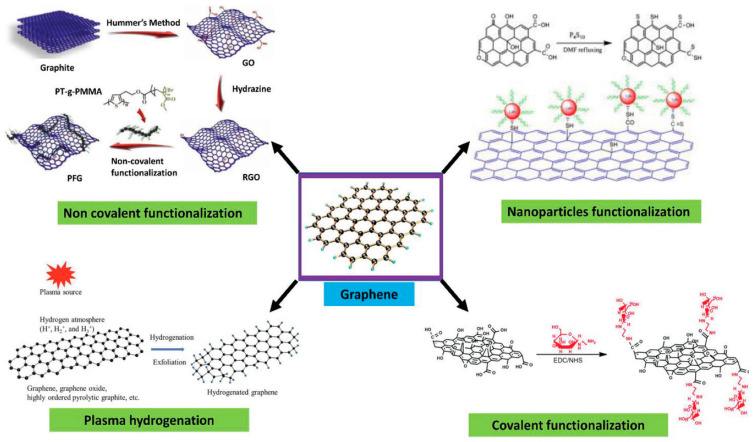
Surface functionalization strategies for graphene-based materials. Reproduced with permission from Ref. [17]. Copyright 2021 Wiley. Licensed under a Creative Commons Attribution (CC BY) License.

In non-covalent surface modification, 1-pyrenebutyric acid N-hydroxysuccinimide ester (PBASE) is often used. Modification through PBASE preserves the electronic properties and planarity of the graphene network without interrupting its sp^2^ bond in its lattice. In covalent modification, the interruption of electronic properties of graphene has been observed [12]. Hwang T.Y et al. [13] constructed an FET biosensor employing PBASE as linker to capture the desired biorecognition molecule, a probe DNA with an amine group at the 5′ end [10]. PBASE acted as a heterobifunctional linker [12,14], serving two different interactions via its two different moieties. The pyrene moiety in PBASE stacked onto the graphene surface through π–π interactions; meanwhile, the succinimide moiety prefers the amino group at the 5′ end in the probe DNA [15]. The same approach was used to immobilize the aptamer and proteins specific in detecting genome DNA or viral antigens in human serum. For example, amino acid residue on the protein surface binds with PBASE through its ester group. Zhuang Hao et al. [16] employed PBASE to immobilize the cytokine aptamer as the biorecognition unit to design a biosensor for detecting cytokine. Facile passivation methods such as non-covalent modification of the graphene surface are promising techniques which introduce Pi-governed molecules such as PASE. Such non-covalent surface modification can be applied to the surface and would allow linker molecules such as PASE to bind to the surface of graphene through π–π interactions. PASE, due to its heterobifunctional characteristic, helps to bind the biorecognition part such as the probe DNA or antibody through its succinimide moiety. The specificity of the graphene-based sensors, therefore, will be increased by the presence of a biorecognition entity as shown in Figure 3.

## 5. The Specificity of DNA-Based Probes

Hwang T.Y et al. [13] employed PBASE to covalently bind the bioreceptors specific to capture SARS-CoV-2, NP and assess the severity of disease using C-reactive protein (CRP). This is achieved through covalent interaction between the carboxylic moiety and the amino group in PBASE and specific antibodies, respectively. This effectively prevents any direct interaction of large molecules with the surface of graphene [13]. There are other methods available to immobilize the biorecognition unit of the biosensor; yet crucially, linker molecules have to be anchored on top of the surface of graphene, giving easy access to the analyte interaction. Passivation of the surface is achieved by linker molecules, commonly by incubating it overnight in an organic solvent, such as dimethylformamide (DMF) [13]. Consequently, the time taken for the incubation of PBASE on top of graphene affects its surface coverage [13,15]. Thus, molecules such as bovine serum albumin (BSA) can be employed to block the surface from non-specific binding of the analyte (SARS-CoV-2 antigens) directly to the surface of the graphene [11]. The specificity of graphene can be increased by introducing bioreceptor molecules that are capable of recognizing target molecules. Bioreceptors boost the sensor’s performance by isolating the signals exclusive to the target molecules (complementary DNA, proteins, peptides, etc.) adhering onto the bioreceptors (probe DNA, antibodies, peptides, etc.), thereby disregarding the signal generated from other molecule interactions.

## 6. Immobilization of the Biorecognition Unit

The attachment of a biorecognition element is carried out by incubating it on a passivated PBASE graphene surface. Previously, the interaction was engineered with the moiety that preferred the -NH_2_ group present in proteins enabling this immobilization method. Lizhou Xu et al. [11] showed that a spike protein of SARS-CoV-2 or an anti-SARS-CoV-2 spike protein S1 monoclonal antibody can be anchored on passivated PBASE (10 mM in DMF) at approximately 2 h in room temperature, followed by addition of a copious amount of 1X PBS to rinse leftover unbound protein molecules [10]. Likewise, the same method was used by Hwang T.Y. et al. [13] but the incubation of PBASE was performed overnight to ensure adherence of PBASE, presenting an increased probability of ssDNA binding onto the surface. Moreover, EDC/NHS was utilized by other research groups [18,19,20], where they pre-activated the carboxylic unit of PBASE. Immobilization of biorecognition molecules could possibly lead to non-specific interactions. Therefore, a blocking strategy is executed right after the introduction of a capture molecule. Bovine serum albumin and EDC/NHS are among the common blocking molecules to investigate the specificity of sensor response towards the analyte alone. Similar strategies were used by Deepshika S. et al. [20] to induce binding of SARS-CoV-2 antigens, solely with immobilized SARS-CoV-2 antibodies.

The choice of biorecognition molecules is crucial depending on target molecules. In the case of SARS-CoV-2 antigens, usually SARS-CoV-2 antibodies are used. Anchoring a biorecognition unit will allow binding interactions to target molecules, as well as forbid any other interaction of molecules in the solution, which can interfere with signal generation and identification. However, the remaining surface uncovered by the biorecognition molecules will enable direct interaction between the sensor and the analyte, which can be reduced by the usage of blocking agents such as polymers, surfactants and DNA [21].

## 7. Detection Mechanism

Construction of a biosensor commonly consists of a receptor (biorecognition) component and a target molecule coupled to a transducer, which transmits electrical or chemical signals to record the interaction. The bioreceptor component complements its target molecule via interactions such as DNA hybridization or other specific interactions. ssDNA or probe DNA are being immobilized onto the surface of graphene to capture target DNA—in this review’s case, SARS-CoV-2—through hybridization. Biological activity or interactions occurring provide a measurable signal, which varies depending on type of biosensor—field effect transistors (FETs) [14,16,22,23,24] and electrochemical biosensors [20,25,26] will provide signals in the form of current or voltage changes. Meanwhile, surface plasmon resonance (SPR)-based biosensors generally provide signals by changes in SPR angle [27,28]. Sensing of SARS-CoV-2 through a FET biosensor can be executed by immobilizing SARS-CoV-2 S1-antibody onto the graphene. Since the antigen–antibody interaction is highly specific, there is a definitive conclusion that the target analyte is present. The graphene surface can provide signals to detect very low levels in the attomolar range due to their highly conductive nature. The doping level on the surface of graphene due to the bulking of target molecule (SARS-CoV-2 antigen) concentration from the lowest concentration to higher concentrations changes the behavior of heterogeneous electron transfer between Ab–Ag bonds. This, in turn, changes the overall electrostatic potential (∆q) in the sensing chamber. FET signals are generally observed by the Dirac point shift and, therefore, the increase in concentration leads to a decrease in Dirac point shift in the current–voltage curve [23].

**Figure 4:**
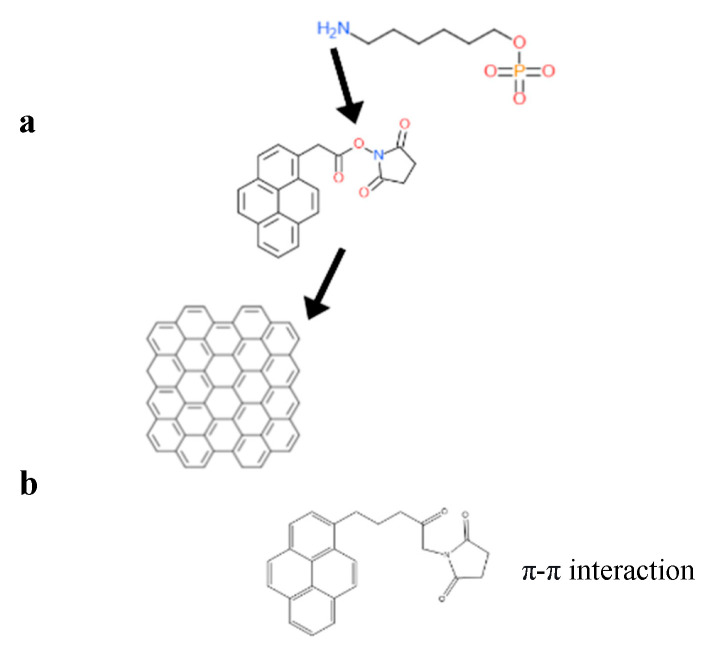
(**a**) The interaction of PBASE and graphene through its pyrene group moiety, while the N-hydroxysuccinimide ester group in PBASE reacts with NH2 group capped at the 5′ end of probe DNA. (**b**) The structure of a heterobifunctional linker, 1-pyrenebutyric acid N-hydroxy-succinimide (PBASE), composed of a pyrene moiety and a N-hydroxysuccinimide ester moiety.

For example, the DNA sequence, with amine capped at the 5′ end (NH2-AACCACACAACCTACTACCTCA-3′), functionalized on the graphene surface through a bifunctional linker, PBASE, is shown in the Figure 4.

## 8. The Field Effect Transistor (FET) Detection Strategy

Nowadays, there is increasing attention towards applying field effect transistors (FETs) as biosensors as the chemical signal can be converted into an electrical signal with open gate structures as front-end transducers [29]. In the 1970s, Bergveld and his co-workers developed the idea of gating the channel of the FET between the charged molecules which are adsorbed onto the surface of a channel by using an electrostatic interaction to examine the proton concentration (pH) of the electrolyte [29,30]. These transistors are called ion-sensitive FETs (ISFETs) and this concept has recently been applied to nanoscale devices such as nanowire (NW) [31,32] and carbon nanotube (CNT) FETs [33,34,35].

Bergveld [29] also accurately predicted that a biochemical interaction with immobilized charged molecules (hydrogels) will give rise to a slew of new sensors. He added that rather than making steady progress in the past 30 years, ISFET sensors have been lagging in practical applications. With the advent of newer interaction moieties, ISFET technology will remain significant for the next 30 years.

To date, analysis techniques reported to detect the presence of chemicals are the quartz crystal microbalance technique [36,37,38], surface-enhanced Raman scattering and surface plasmon resonance [39,40], electrochemical sensing [41,42], microfluidic devices [43], and field effect transistor (FET) build biosensors [44,45]. For producing better results, the best methods are FET build devices and electrochemistry techniques because of their extreme sensitivity, power efficiency, rapidness, compactness, infallibility, flexibility and inexpensiveness.

For producing label-free detection of biomolecules, usage of the bio-FETs in the biosensor industry has been resurging due to researchers’ initiative in identifying and utilizing several target molecules of interest. Perhaps there are various reports on the high electron mobility transistor (HEMT) type of FET sensor for chemical and biological applications [46,47]. Trans et al. [34] and Kim et al. [48] have developed and demonstrated the usage of carbon nanotubes (CNTs) as the channels in CNT-FETs, and investigated biotin protein complexes by extended-gate FET-based biosensors for the detection of streptavidin. In the device channel, the carrier density is mainly controlled by gating, realized by an electrostatic potential V*_lg_* applied at the adjacent liquid containing the analytes (liquid gating) or through an electrostatic potential V*_bg_* applied to the back electrode beneath the channel (back gating). Here, the analyte must be highly dispersed in a buffer solution and should be conductive.

Using a FET arrangement, most specific species are generally sensed by graphene sensors (biosensors). The foremost characteristic for the detection of these species depends on the resistivity on back-gate voltage, which shows a peak that relates to a charge neutrality point (CNP peak) and this point is defined by a fermi level crossing the Dirac point, where the total charge in the graphene has to be zero. Preferably, the shift in the CNP peak in sensors is dependent on the doping caused by the adsorbed molecules which are being detected. Nevertheless, there is a hysteresis observed in the water and atmospheric solution which are at different positions of the CNP peak during the downward and upward sweeping of the back-gate voltage in the real graphene sensors for FET design. Such peak shifting behavior is often recognized as being due to diffusion and charge trapping inside the gate isolated layer (e.g., positive/negative bias temperature instability and ion diffusion).

Applications of FET-based biosensors necessitate examining large numbers of patient RNA or DNA samples, or multiple SNVs. FET-based biosensors are used to quantify and identify the biological analytes in which target binding is directly transduced into changes for high-sensitivity detection in FET conductance [49,50,51,52,53]. The fabricated transistor surfaces are amended with receptors (e.g., antibodies, nucleic acids, and proteins) to allow the selective recognition of molecular types of targets, ranging from proteins and [54] nucleic acids [55,56] to small molecules [57].

**Figure 5:**
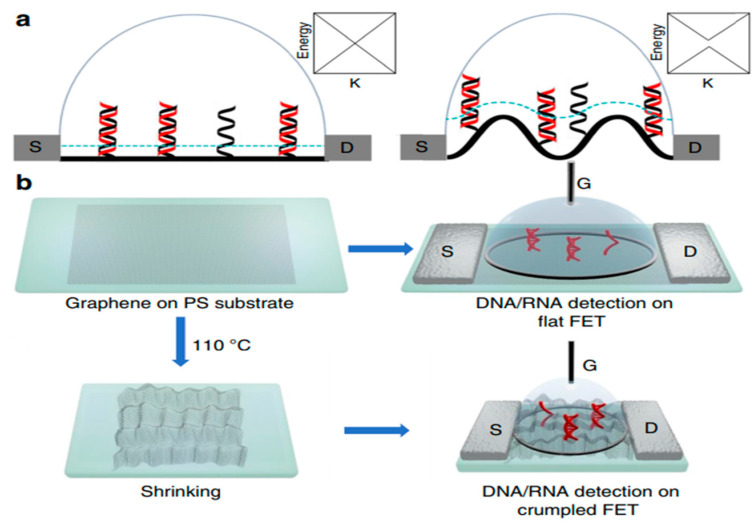
Scheme and characterization of (**a**) flat and (**b**) crumpled graphene FET biosensor. Reproduced with permission from Ref. [13]. Copyright 2020 Springer Nature. Licensed under a Creative Commons Attribution (CC BY) License.

In this review, field effect transistor-type biosensors have been highlighted to give researchers working in this field an overview of their recent advances as sensitive, label-free, and selective electronic biosensors as point-of-care devices specifically for clinical applications. FET-based biosensors will play a role in monitoring the real-time progress of pandemics and diseases such as cancer, HIV, and CVDs, paving the way for technological breakthroughs in rapid screening, thereby assisting in the prevention of spread. However, there are some fundamental challenges in the development of FET-based biosensors. On the outset, FET-based biosensors, as shown in Figure 5, have initiated a diagnostic revolution for the upcoming generation.

## 9. Electrochemical Detection Technique

**Figure 6:**
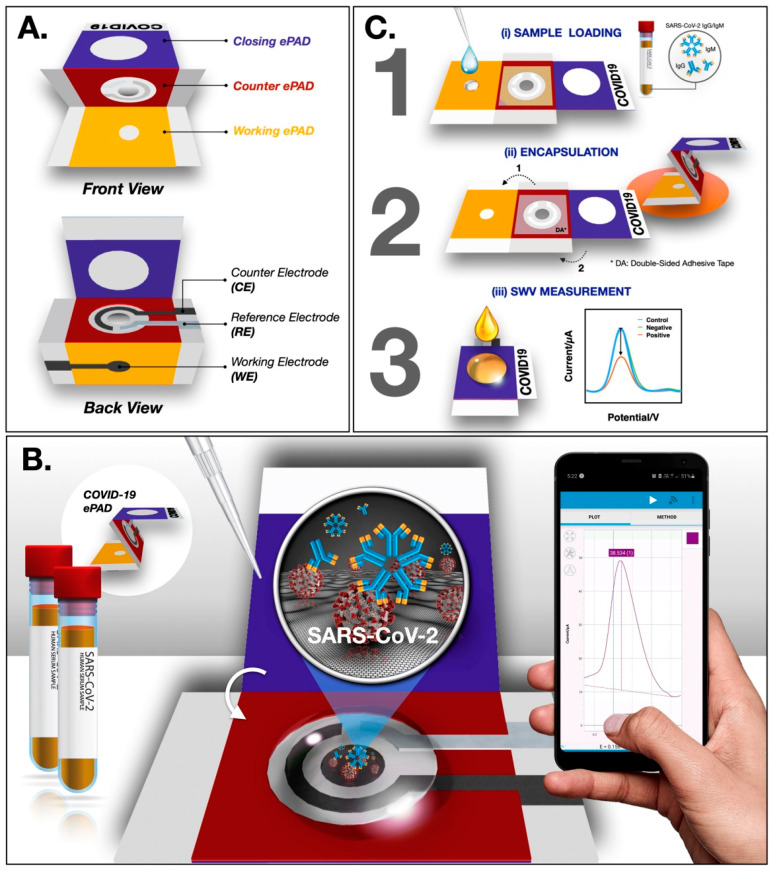
Schematic representation of ePAD fabrication and operation (**A**) Design of the ePAD (**B**) Interface design between ePAD and mobile phone (**C**) Operational schematic of the ePAD. Reproduced with permission from Ref. [2]. Copyright 2021Elsevier.

In a recent study, Yakoh et al. [2] developed a paper-based biosensor for the electrochemical detection of COVID-19. The ePAD was fabricated though a wax printer and consists of three layers—the counter, closing and working layers. In the working ePAD zone, the GO solution is deposited for the operation. Finally, the COVID-19 ePAD is probed for the detection of spike proteins of SARS-CoV-2 (Figure 6). Upon investigation, a faster response and a greater LOD of 1 ng/mL are achieved using the ePAD, which is superior to a traditional colorimetric lateral flow assay.

**Figure 7:**
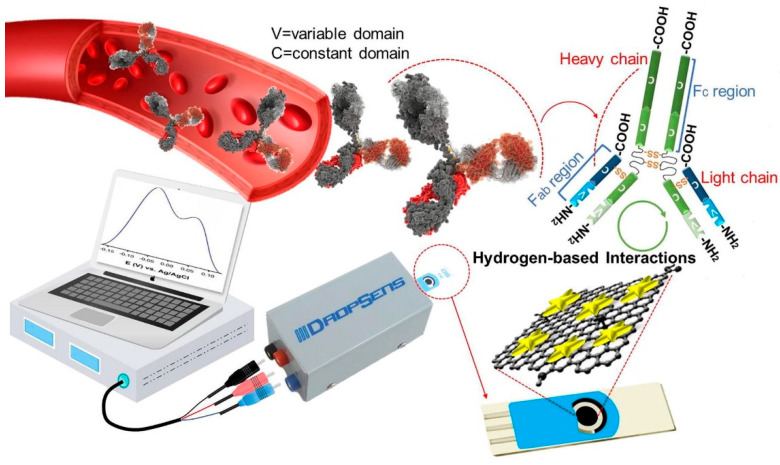
Electrochemical detection of IgG antibody through GO/Au nanostar composites. Reproduced with permission from Ref. [3]. Copyright 2021 Elsevier.

Seyyed et al. [3] developed a highly sensitive label-free biosensor for the detection of IgG antibodies of SARS-CoV-2 in blood samples. The fabricated electrochemical biosensor is based on graphene/Au nanostar composites. The composites are loaded in the working electrode area of screen-printed carbon electrodes (SPCEs) and utilized for analysis. The authors activated the GO with 8-hydroxyqyuinoline (8H), 1-ethyl-3-(3-dimethylaminopropyl), carbodiimide (EDC) and N-hydroxy succinimide (NHS). These functional groups present in the GO help in the detection of IgG antibodies. Graphene oxide typically provides a high specific surface area with numerous hydrophilic functional groups such as –OH and C=O and this helps in the interaction with the target analyte. The incorporation of 8H into GO enhances the overall rate of functional groups (-OH), which, in turn, boosts the performance of the sensor with its quinolone structure. Further, the introduction of NHS and EDC to GO/8H system leads to carbonyl and hydrogen-based functional group activation. This activation of GO is beneficial in the efficient absorption of IgG via electroactive functional groups (amines). Ultimately, the surface interaction in the working electrode possessing activated GO can be seen as a voltametric response. The electrical conductivity and electrochemical activity of activated GO are boosted by forming a composite with Au nanostars. The overall process is presented in Figure 7.

**Figure 8:**
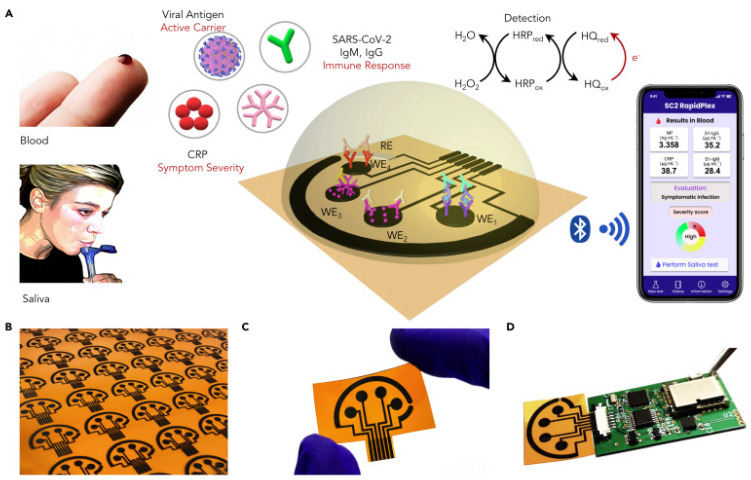
Wireless graphene-based multiplexed telemedicine platform for electrochemical detec-tion of COVID-19 biomarkers from blood/saliva (**A**) Schematic illustration of the SARS-CoV-2 RapidPlex multisensor telemedicine platform for detection of SARS-CoV-2 viral proteins, anti-bodies (IgG and IgM), and inflammatory biomarker C-reactive protein (CRP). Data can be wire-lessly transmitted to a mobile user interface. WE, working electrode; CE, counter electrode; RE, reference electrode (**B**) Mass-producible laser-engraved graphene sensor arrays (**C**) Photograph of a disposable and flexible graphene array (**D**) Image of a SARS-CoV-2 RapidPlex system with a graphene sensor array connected to a printed circuit board for signal processing and wireless communication. Reproduced with permission from Ref. [15]. Copyright 2020 Cell Press, Else-vier.

In an interesting work from Wei Gao research group, they developed a wireless graphene-based multiplexed telemedicine platform for electrochemical detection of COVID-19 biomarkers from blood/saliva. The platform was named SARS-CoV-2 RapidPlex. SARS-CoV-2 RapidPlex consists of four graphene working electrodes, a reference electrode (Ag/Ag/Cl) and a graphene counter electrode. All these electrodes are patterned on polyimide (PI) through CO_2_ laser engraving. The activity of SARS-CoV-2 RapidPlex is shown in Figure 8.

**Figure 9:**
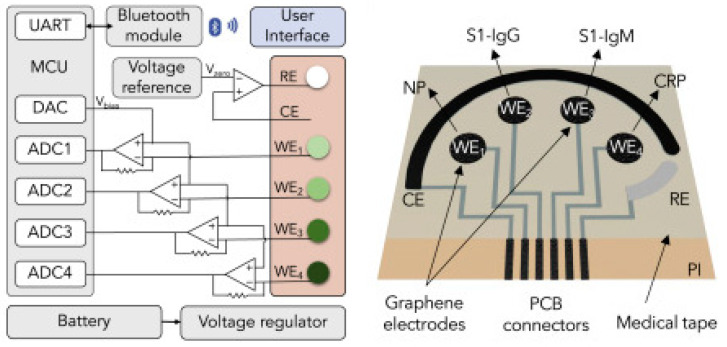
Block diagram of the RapidFlex sensing platform with interface is shown in the left and the sensor array layout is shown on the right. Reproduced with permission from Ref. [15]. Copyright 2020 Cell Press, Elsevier.

The block diagram and sensor array layout of SARS-CoV-2 RapidPlex are displayed in Figure 9. With the fabricated device, the authors have successfully demonstrated the detection of COVID-19 antibodies from blood and saliva samples wirelessly [15].

## 10. Comparison between Various Detection Techniques

Although there are several techniques available for detecting macromolecules of interest, graphene-based FETs provide a unique platform to analyze very low concentrations at the attomolar and zeptomolar ranges with repeatable results. The other methods listed in Table 1 include an electrochemical-based approach, an SERS-based approach and surface plasmon resonance-based methodologies but the speed at which FET-based sensors provide a signal is the fastest.

## 11. Conclusions

This review has presented the possibility of usage of two-dimensional (2D) graphene materials in detection of microbial analytes at low concentrations. Both graphene, which is a hexagonally arranged carbon atom with a single-atom thickness, as well as graphene oxide (GO), which is the oxidized variant of graphene, have suitable qualities to detect low concentrations of analytes at the attomolar and zeptomolar ranges. Reduced graphene oxide (rGO), which is produced upon eliminating the oxygen groups by reducing agents, belongs to the graphene family of materials (GFM), with exceptional value for acting as the base material for designing sensors. Graphene exhibits a high surface to volume ratio due to its single layer of carbon atom arrangement. This directly aids in realizing a single molecular detection of disease biomarkers [58]. In other terms, even when a single viral protein comes under contact with the graphene surface, it can be accurately detected. This makes graphene an ideal candidate for fabricating a biosensor for disease monitoring. The functional groups, such as hydroxyl, epoxide, carbonyl, and carboxy groups, in GO make its surface significantly more hydrophilic than that of its counter parts, rGO and graphene. In addition, surface oxygen helps in chemisorption or functionalization with enzymes, proteins and DNA/RNA. Through highly selective functionalization of GFM, specific biomarker analytes can be targeted and detected. As synthetic recognition constructs such as monoclonal antibodies and probe DNAs can be rapidly manufactured, they can serve as effective biorecognition units for highly specific interactions with the SARS-CoV-2 virus. With graphene’s unique conductive properties, the biosensors fabricated from the complex can be used in a label-free detection strategy, which can, in turn, enable large studies to be undertaken in line with the current pandemic.

## Figures and Tables

**Table 1:** An overview on recently reported methods for the determination of macromolecules using various sensors.

S No.	Device	Surface Functionalization	Analyte	LOD	Reference
1	Paper-Based Electrochemical Biosensor	GO-spike protein receptor-binding domain (SP RBD)- SARS-CoV-2	SARS-CoV-2 IgG/SARS-CoV-2 IgM	SARS-CoV-2 IgG (0.96 ng/mL) or SARS-CoV-2 IgM (0.14 ng/mL)	[2]
2	Graphene-Based Electrochemical Biosensor	Au NS with an activated graphene	Monoclonal IgG antibody of SARS-CoV-2′s S1 protein	0.18 × 10−19% V/V	[3]
3	Graphene Aptasensor	PBASE-Aptamer-SARS-CoV-2	SARS-CoV-2	160 aM for COVID-19 neutralizing antibodies in serum	[11]
4	Aptameric GFET	PBASE-Aptamer-Cytokine (IL or TNF or IFN)	Cytokine	476 × 10−15 M (IFN-Υ), 608 × 10−15 M (IL-6), or 611 × 10−15 M (TNF-α)	[16]
5	Deformed Graphene Channel-GFET	PBASE-*p*DNA-*t*DNA	DNA/RNA	600 zM (~18 molecules)	[13]

## Data Availability

Data Sharing not applicable.
